# Atomic-level insights in optimizing reaction paths for hydroformylation reaction over Rh/CoO single-atom catalyst

**DOI:** 10.1038/ncomms14036

**Published:** 2016-12-22

**Authors:** Liangbing Wang, Wenbo Zhang, Shenpeng Wang, Zehua Gao, Zhiheng Luo, Xu Wang, Rui Zeng, Aowen Li, Hongliang Li, Menglin Wang, Xusheng Zheng, Junfa Zhu, Wenhua Zhang, Chao Ma, Rui Si, Jie Zeng

**Affiliations:** 1 Hefei National Laboratory for Physical Sciences at the Microscale, Key Laboratory of Strongly-Coupled Quantum Matter Physics of Chinese Academy of Sciences, Hefei Science Center & National Synchrotron Radiation Laboratory, Department of Chemical Physics, University of Science and Technology of China, Hefei 230026, China; 2 Shanghai Synchrotron Radiation Facility, Shanghai Institute of Applied Physics, Chinese Academy of Sciences, Shanghai 201204, China

## Abstract

Rh-based heterogeneous catalysts generally have limited selectivity relative to their homogeneous counterparts in hydroformylation reactions despite of the convenience of catalyst separation in heterogeneous catalysis. Here, we develop CoO-supported Rh single-atom catalysts (Rh/CoO) with remarkable activity and selectivity towards propene hydroformylation. By increasing Rh mass loading, isolated Rh atoms switch to aggregated clusters of different atomicity. During the hydroformylation, Rh/CoO achieves the optimal selectivity of 94.4% for butyraldehyde and the highest turnover frequency number of 2,065 h^−1^ among the obtained atomic-scale Rh-based catalysts. Mechanistic studies reveal that a structural reconstruction of Rh single atoms in Rh/CoO occurs during the catalytic process, facilitating the adsorption and activation of reactants. In kinetic view, linear products are determined as the dominating products by analysing reaction paths deriving from the two most stable co-adsorbed configurations. As a bridge of homogeneous and heterogeneous catalysis, single-atom catalysts can be potentially applied in other industrial reactions.

## Abstract (web summary)

Despite the advantages of using heterogeneous catalysts, most successful rhodium hydrogenations are carried out with homogeneous catalysts. Here the authors report a supported single atom rhodium catalyst providing high activities and selectivities for propene hydroformylation.

Hydroformylation of olefins is one of the most important industrial processes because its corresponding product, aldehydes, can be widely applied as intermediates for synthetic esters, alcohols, carboxylic acids, aliphatic amines and other fine chemicals^1^,^2^. Rh-based homogeneous catalysts are commonly used in this reaction, and have revealed remarkable activity and selectivity^1^,^2^. Compared with homogeneous catalysts, heterogeneous counterparts show lower selectivity, but can be more facilely separated from the reaction mixture^3^,^4^,^5^,^6^. To combine the advantages of heterogeneous and homogeneous catalysts, immobilizing Rh-based species onto solid supports is an efficient approach^7^,^8^,^9^,^10^. However, the relatively low stability of the physical adsorption will induce the leaching of active species, including the organic ligands and the metal species. Therefore, developing efficient heterogeneous catalysts for hydroformylation reactions is of high importance, and remains as a grand challenge.

Single-atom catalysts are a class of heterogeneous catalysts recently developed, in which isolated metal atoms are singly dispersed on supports^11^,^12^. Owing to significantly high efficiency of metal atom usage, low-coordination environment of metal centers, and strong metal-support interaction, single-atom catalysts have revealed remarkable activity in a variety of reactions, including oxidation, water gas shift and hydrogenation^11^,^12^,^13^,^14^,^15^,^16^,^17^,^18^,^19^,^20^,^21^,^22^,^23^,^24^,^25^,^26^,^27^,^28^,^29^. More importantly, when the size of metal catalysts is reduced into single-atom scale, the selectivity in certain reactions is much different compared with their nanocrystal or nanocluster counterparts^27^,^28^,^29^. This transformation in selectivity probably originates from spatially uniform single-atom active sites and charge transfer between metal atoms and supports^27^,^28^,^29^. Therefore, as the bridge between homogeneous and traditional heterogeneous catalysts, single-atom catalysts are expected to serve as desired catalysts for hydroformylation reactions with both high activity and selectivity.

Herein, we report the preparation of CoO-supported Rh single-atom catalysts (Rh/CoO) with remarkable catalytic activity and chemoselectivity towards hydroformylation reactions. By increasing Rh mass loading, isolated Rh atoms switched to aggregated nanoclusters of different atomicity. During the hydroformylation of propene, Rh/CoO achieved the highest turnover frequency (TOF) number of 2,065 h^−1^ and selectivity of 94.4% for butyraldehyde among the obtained atomic-scale Rh-based nanocatalysts. In addition, 95.8% of the original reaction activity for Rh/CoO was preserved with the selectivity for butyraldehyde maintaining at 94.0% after five rounds of reaction, indicating their noticeable stability. Further mechanistic studies revealed that a structural reconstruction of Rh single atoms in Rh/CoO occurred during the catalytic process, facilitating the adsorption and activation of reactants. The kinetic analysis indicates the dominating products of linear products based on the analysis of reaction paths deriving from the two most stable co-adsorbed configurations.

## Results

### Synthesis and structural characterizations

**Figure 1:**
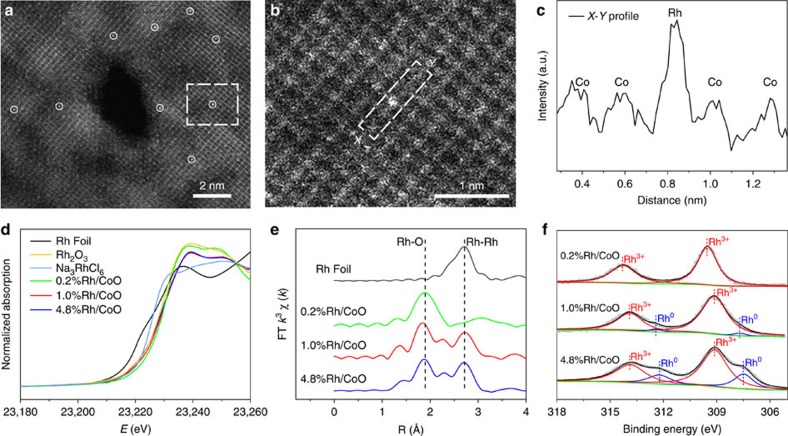
Structural characterization of Rh/CoO with different Rh loadings. (**a**) HAADF-STEM image of 0.2%Rh/CoO single-atom structures. Rh single atoms marked in white circles are uniformly dispersed on the CoO nanosheets and occupy exactly the positions of Co atoms. (**b**) Magnified HAADF-STEM image of 0.2%Rh/CoO corresponding to the area of the white square in **a**. (**c**) Intensity profile along the line *X*–*Y* in the magnified HAADF-STEM image (**b**). (**d**) Rh K-edge XANES profiles for Rh/CoO with different Rh loadings. (**e**) Rh K-edge EXAFS spectra in *R* space for Rh/CoO with different Rh loadings. (**f**) XPS spectra of Rh/CoO with different Rh loadings.

To begin with, atomically thin CoO nanosheets were synthesized based on a previously reported method^30^. The obtained CoO nanosheets took a face-centered cubic (*fcc*) crystalline nature in high purity and uniformity (Supplementary Fig. 1). In a typical synthesis of Rh/CoO, Na_3_RhCl_6_ solution (10 mM, 0.19 ml) was injected into the aqueous solution containing CoO nanosheets (100 mg), followed by stirring at room temperature for 4 h. In this process, Rh atoms replaced Co atoms on the surface of CoO nanosheets via the galvanic replacement reaction between the precursor and substrate. Figure 1a shows a high-angle-annular-dark-field scanning transmission electron microscopy (HAADF-STEM) image of Rh/CoO. As revealed by bright spots and marked by white circles, individual Rh atoms were dispersed on the surface of CoO nanosheets. Further observation in the magnified HAADF-STEM of Rh/CoO determines that Rh atoms exactly occupied the positions of the Co atoms (Fig. 1b). The existence of Rh single atoms was further verified by the intensity profile (Fig. 1c) along the line *X–Y* (Fig. 1b) in the observed image, as the signal intensity is approximately proportional to the square of atomic number^31^. Inductively coupled plasma-atomic emission spectrometry (ICP-AES) result indicates that the mass loading of Rh on CoO is 0.2%, which corresponds to the amount of precursors. For simplification, we donated the synthesized Rh single atoms dispersed on CoO structures as 0.2%Rh/CoO. In addition, we measured the leached Co species by ICP-AES. The molar ratio of leached Co species to added Rh single atoms was determined as 1.41. In other words, two surface Co atoms were replaced with two Rh atoms on CoO nanosheets, and a Co vacancy was involved. When the amount of Na_3_RhCl_6_ solution (10 mM) increased to 0.97 and 4.85 ml, 1.0%Rh/CoO and 4.8%Rh/CoO were obtained, where the mass loadings of Rh were 1.0 and 4.8%, respectively. Supplementary Fig. 2 reveals the HAADF-STEM images of 1.0%Rh/CoO and 4.8%Rh/CoO. Besides Rh single atoms, subnanometer Rh clusters containing about tens of atoms were also generated in the synthesis of 1.0%Rh/CoO. As for 4.8%Rh/CoO, Rh nanoclusters with the size of 1.5–2.0 nm dominated the configuration of Rh species. This morphological variation from single atoms, subnanometer clusters to nanoclusters is verified by X-ray diffraction patterns of Rh/CoO with different Rh mass loadings (Supplementary Fig. 3). Every peak in 0.2%Rh/CoO could be assigned to *fcc* CoO without the signal of Rh species, because of single-atom structures and extremely low mass loading. In 1.0%Rh/CoO and 4.8%Rh/CoO, the peaks of *fcc* Rh were observed due to the existence of Rh clusters. In addition, Rh single atoms supported on bulk CoO, denoted as Rh/*bulk*-CoO, were also synthesized based on similar procedures except for using different CoO supports (Supplementary Fig. 4).

The X-ray absorption near-edge spectroscopy (XANES) and extended X-ray fine structure (EXAFS) were measured to determine the electronic and local structures of chemical nature of the samples. The Rh K-edge XANES profiles in Fig. 1d indicate that Rh species in 0.2%Rh/CoO were in a higher oxidation state than those in 1.0%Rh/CoO and 4.8%Rh/CoO, according to the higher energy for absorption edge and the stronger intensity for white line. It is worth noting that the spectrum of 1.0%Rh/CoO was almost identical to that of 4.8%Rh/CoO. As shown in EXAFS in *R* space (Fig. 1e), 0.2%Rh/CoO exhibited a prominent peak at ca. 2.0 Å from the Rh-O shell with a coordination number (*CN*) of 5.8 (Supplementary Table 1). No other typical peaks for Rh–Rh or Rh-Cl contribution at longer distances (> 2.0 Å) were observed. Thus, Rh atoms were atomically dispersed throughout the whole 0.2%Rh/CoO without contour ions. When the Rh loading was increased to 1.0 and 4.8%, a new peak located at ca. 2.7 Å was observed, implying that the Rh–Rh metallic bonding started to form (Fig. 1e). Moreover, the increased Rh loadings lowered Rh-O coordination to 4.6 and 4.1 for 1.0%Rh/CoO and 4.8%Rh/CoO, respectively (Supplementary Table 1). Their Rh–Rh *CN*s, however, were relatively low (*CN*<3.0) compared with that of Rh foil (*CN*=12.0) (Supplementary Table 1), indicating the formation of very small (<1.0 nm) Rh clusters^32^. This result was in good agreement with HAADF-STEM images (Supplementary Fig. 2), where the average sizes of Rh clusters for both samples were below 2.0 nm. To further characterize the electronic properties of Rh/CoO, we conducted X-ray photoelectron spectroscopy (XPS) measurements. As shown in Fig. 1f, the binding energy of Rh 3*d*_5/2_ in 0.2%Rh/CoO was determined as 309.5 eV, which indicated the existence of Rh^3+^ state for Rh single atoms. As for 1.0%Rh/CoO and 4.8%Rh/CoO, the binding energies of Rh 3*d*_5/2_ contributed from Rh^3+^ state slightly decreased to 309.2 and 309.1 eV, respectively. Moreover, new Rh 3*d*_5/2_ peaks at 307.7 and 307.5 eV were observed for 1.0%Rh/CoO and 4.8%Rh/CoO, respectively, both of which were assigned to the metallic state of Rh. These results corresponded well to the observation in HAADF-STEM images of both the samples where Rh clusters were generated (Supplementary Fig. 2).

### Catalytic performance in hydroformylation reaction

**Figure 2:**
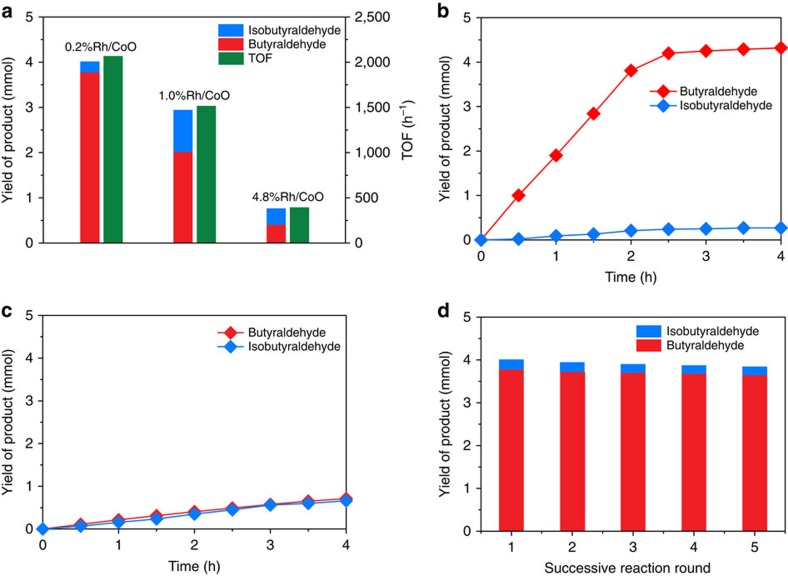
Catalytic performance in the hydroformylation of propene. (**a**) TOFs and product yields of 0.2%Rh/CoO, 1.0%Rh/CoO, and 4.8%Rh/CoO in the hydroformylation of propene at 100 °C after 2 h. (**b**,**c**) Time courses of the hydroformylation of propene over 0.2%Rh/CoO and 4.8%Rh/CoO, respectively, at 100 °C. (**d**) Product yields of 0.2%Rh/CoO over the course of five rounds of successive reaction.

We applied the established atomic-scale Rh-based catalysts in the hydroformylation of propene to evaluate their catalytic performance. A blank test was performed with only CoO nanosheets added, in which no product was observed. Figure 2a illustrates the product yields of CoO-supported Rh catalysts with different mass loadings. Under the same reaction condition (100 °C, 2 h), the TOF number of 0.2%Rh/CoO reached 2065, h^−1^, 1.4 and 5.2 times as high as that of 1.0%Rh/CoO and 4.8%Rh/CoO, respectively. Moreover, the selectivity for butyraldehyde over 0.2%Rh/CoO reached 94.4%, while only 68.7 and 53.9% of butyraldehyde were generated in the products over 1.0%Rh/CoO and 4.8%Rh/CoO, respectively. This decreasing trend of selectivity against mass loadings was further reviewed by reaction profiles. Nowadays, more than 75% of all hydroformylation reactions are based on Rh triarylphosphine catalysts^2^. For Rh triarylphosphine catalysts, the TOF number is ca. 5000, h^−1^, and the selectivity for linear products reaches >90%. Thus, 0.2%Rh/CoO exhibited comparable catalytic activity and selectivity to Rh-based homogeneous catalysts. As shown in Fig. 2b, the conversion of propene was increased to 92.2% after 2.5 h of reaction over 0.2%Rh/CoO, during which the selectivity for butyraldehyde maintained at 94.4%. Further prolonging the reaction time to 4 h led to a slight increase in conversion to 94.6% without any change in selectivity. By comparison, when 4.8%Rh/CoO was utilized instead, the conversion only reached 27.4% after 4 h, with selectivity for butyraldehyde of 51.8% (Fig. 2c). It is worth noting that Rh single atoms supported on bulk CoO exhibited similar catalytic properties to those of 0.2%Rh/CoO (Supplementary Fig. 5). In addition, the stability of 0.2%Rh/CoO was also studied by performing successive rounds of reaction, as shown in Fig. 2d. After five rounds, 95.8% of the original reaction activity was preserved with the selectivity for butyraldehyde maintaining at 94.0%. According to EXAFS results, the state of Rh species was barely changed in the reuse test (Supplementary Fig. 6). ICP-AES data further confirms the high stability of Rh single atoms supported on CoO by showing that 96.1% of Rh species were preserved (Supplementary Table 2). The high stability of 0.2%Rh/CoO is of high importance for potential applications in industrial processes by reducing the cost and pollution efficiently. After the catalytic test over 0.2%Rh/CoO, the catalysts were removed by centrifugation. Afterwards, we conducted catalytic tests by using supernatant as catalysts. The conversion of propene was below 0.5%, with the selectivity for butyraldehyde of ∼54%. Therefore, the catalytic activity was dominantly contributed by Rh single atoms rather than the leached Rh species. In addition, five more types of substrates including ethylene, heptylene, nonylene, decene and styrene were catalysed by 0.2%Rh/CoO for hydroformylation reactions. After the reactions proceeded over 0.2%Rh/CoO at 100 °C for 5 h, the conversions were above 90% with the selectivity for linear products exceeding 85% (Supplementary Table 3).

### Interaction between reactants and 0.2%Rh/CoO

**Figure 3:**
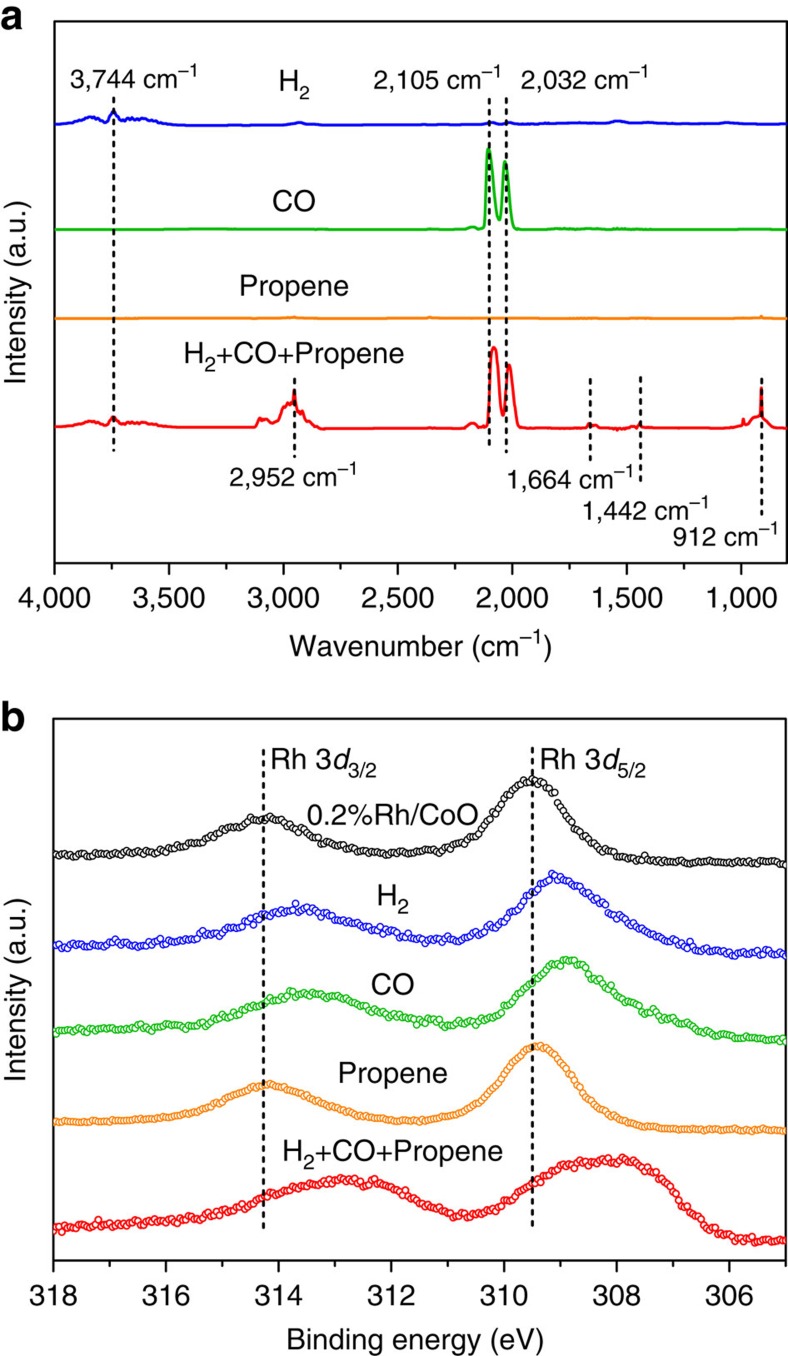
The interaction between reactants and 0.2%Rh/CoO. (**a**) *In situ* DRIFT spectra of 0.2%Rh/CoO after the treatment of the sample with different gas at 100 °C. The peak at 3,744 cm^−1^ corresponds to the stretching vibration of –OH group; the peaks at 2,105 and 2,032 cm^−1^ correspond to the stretching vibrations of CO adsorbed on Co and Rh atoms, respectively; the peaks at 2,952, 1,664, 1,442 and 912 cm^−1^ correspond to the stretching vibration of C-H, the stretching vibration of C=C, the scissoring vibration of C–H, and the bending vibration of C–H in propene, respectively. (**b**) *In situ* XPS spectra of 0.2%Rh/CoO before and after the treatment of the sample with different gas at 100 °C.

We conducted *in situ* diffuse reflectance infrared Fourier transform (DRIFT) and *in situ* XPS measurements to investigate the adsorption properties of reactants on the surface of 0.2%Rh/CoO. In order to simulate the reaction conditions, 0.2%Rh/CoO was respectively exposed to H_2_, CO, propene, and the mixed gas (H_2_:CO:propene=1:1:1) at 100 °C, followed by measurements of *in situ* DRIFT (Fig. 3a). The peak located at 3,744 cm^−1^ for the sample after the treatment with H_2_ corresponded to the stretching vibration of –OH, indicating that the dissociated H atoms over Rh single atoms adsorbed on the nearby O atoms. The exposure of the sample to CO gave rise to the appearance of two peaks at 2,105 and 2,032 cm^−1^, which were assigned to the stretching vibrations of adsorbed CO on Co and Rh atoms, respectively. When the sample was treated with propene, no obvious peaks were detected, indicating the weak adsorption of propene on 0.2%Rh/CoO. Interestingly, the peaks of propene were clearly observed after the treatment of 0.2%Rh/CoO with the mixed gas in *in situ* DRIFT profile. As such, the adsorption of propene was significantly facilitated in the atmosphere containing both H_2_ and CO. We speculate that the enhanced adsorption of propene derives from the structural reconstruction of Rh single atoms with the help of both H_2_ and CO. In addition, the peak for the stretching vibration of adsorbed CO on Rh atoms red-shifted to 2,014 cm^−1^ in the atmosphere of the mixed gas, 18 cm^−1^ lower than that in the CO atmosphere. This red-shift indicates a stronger interaction between CO and Rh single atoms after the treatment of 0.2%Rh/CoO with the mixed gas, compared with that after the exposure to only CO.

The adsorption properties of reactants were also revealed by *in situ* XPS measurements. *In situ* XPS measurements were conducted after the treatment of 0.2%Rh/CoO with different gas at 100 °C in a high pressure and high temperature reaction cell attached to the XPS end-station. When the sample was exposed to H_2_ or CO gas at 100 °C, the binding energies of Rh 3*d* for 0.2%Rh/CoO were 0.4 and 0.6 eV lower than that without the exposure to any gas, respectively (Fig. 3b). In contrast, the variation in binding energies of Rh 3*d* after the treatment of 0.2%Rh/CoO with propene was negligible (Fig. 3b). Accordingly, the interaction between Rh single atoms and propene was much weaker than that with CO or H_2_. In the atmosphere of the mixed gas, the binding energy of Rh 3*d* was 1.3 eV lower relative to that without any gas treatment (Fig. 3b). This deviation was even larger than that in the atmosphere of H_2_ or CO, indicating that the exposure of the sample to the mixed gas strengthened the interaction between Rh single atoms and reactants compared with that treated by sole gas. The *in situ* XPS results corresponded well to the *in situ* DRIFT analysis.

### DFT studies

**Figure 4:**
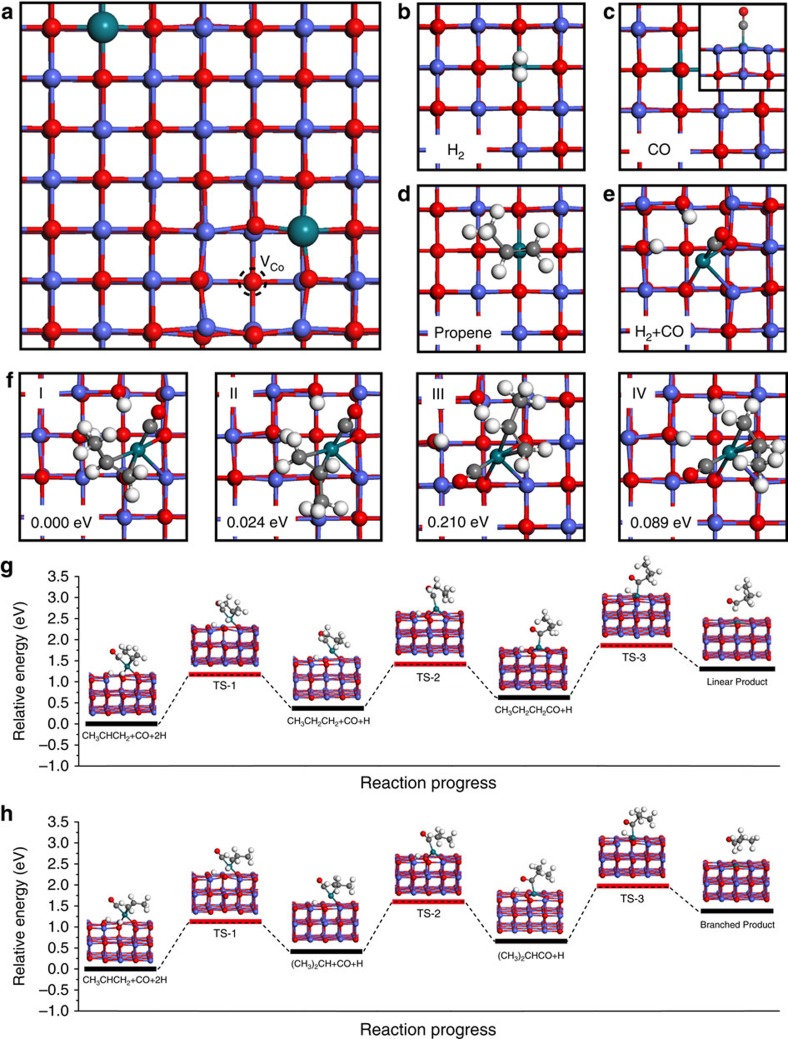
DFT calculations of Rh_1_/CoO in the hydroformylation of propene. (**a**) The model of Rh_1_/CoO. Two Rh atoms occupy the position of two Co atoms, together with the involvement of a Co vacancy. (**b**–**d**) Top views of the adsorption configurations of H_2_, CO, and propene on Rh_1_/CoO, respectively. (**e**) Top view of the co-adsorbed configuration of both H_2_ and CO. (**f**) Four co-adsorbed configurations of reactants and their corresponding adsorption energies. The most stable configuration I was taken as the energy standard (Δ*E*=0.000 eV). Adsorption energies of configuration II, III and IV were 0.024, 0.210 and 0.089 eV, respectively. (**g**,**h**) Reaction paths deriving from configuration I and II, respectively.

To rationalize the remarkable catalytic properties of 0.2%Rh/CoO in hydroformylation of propene, we carried out density functional theory (DFT) calculations. The model was established by replacing two surface Co atoms with two Rh atoms on a CoO thin film and involving a Co vacancy (Fig. 4a). This model was named as Rh_1_/CoO. First-principle simulations were performed to calculate the adsorption energies of H_2_, CO, and propene on Rh_1_/CoO, as shown in Fig. 4 and Supplementary Table 4. When H_2_, CO, or propene adsorbed on Rh_1_/CoO, CO was endowed with the highest adsorption energy of −1.41 eV (Fig. 4b–d). Although the adsorption energy of H_2_ on Rh_1_/CoO was as low as −0.02 eV, the spontaneous dissociation of H_2_ over Rh atoms still enabled a strong interaction between H_2_ and Rh_1_/CoO. The dissociated H atoms adsorbed on O atoms nearby Rh single atoms to form –OH groups. This result was well consistent with our *in situ* DRIFT results. As for propene, the low adsorption energy of −0.46 eV led to the weak adsorption of propene on Rh_1_/CoO. Notably, the position of Rh single atoms did not change during the adsorption of these reactants.

Due to relatively strong interaction with Rh single atoms, CO was regarded to preferentially occupy the active sites on the surface of CoO supports. This process was followed by the adsorption and dissociation of H_2_ (Fig. 4e). Interestingly, Rh single atoms moved after the adsorption of both H_2_ and CO on Rh_1_/CoO. Supplementary Fig. 7a shows the in-plane displacement of the Rh single atom which deviated from the lattice point by 1.1 Å towards the center of the unit cell. In addition, the Rh single atom moved ca. 1.3 Å out of the plane (Supplementary Fig. 7b). In other words, structural reconstruction of Rh single atoms occurred in the atmosphere containing both H_2_ and CO. The reconstruction of active Rh atoms facilitated the adsorption of propene, in that the adsorption energy of propene significantly increased to −0.80 eV (Supplementary Table 4). The enhanced adsorption of propene was directly verified by both *in situ* DRIFT and *in situ* XPS measurements. In addition, the interaction between CO and Rh single atoms was also strengthened in the co-adsorbed configurations. The bond length of CO slightly increased to 1.17 Å, while it was 1.16 Å when only CO adsorbed on Rh_1_/CoO. The elongation of bond lengths was able to induce red-shift of stretching vibration frequency for adsorbed CO on Rh atoms, well consistent with the observation in *in situ* DRIFT measurements.

As a result, co-adsorbed configurations of reactants and catalysts were established. On the basis of DFT calculations, four stable configurations, respectively donated as configuration I, II, III, and IV were proposed as shown in Fig. 4f. When the most stable configuration I was taken as the energy standard (Δ*E*=0.000 eV), adsorption energies of configuration II, III, and IV were calculated to be 0.024, 0.210, and 0.089 eV, respectively. The hydroformylation reaction occurs when a H atom attacks one end of C=C bond in olefin, followed by the addition of CO in another end of C=C bond and at last the other hydrogen atom combines with the C atom in C=O as shown in Fig. 4. Therefore, the site of H atom added directly determines the selectivity of products (that is, linear products or branched products). When Rh nanocrystals were involved in this reaction, H atoms could attack propene in any directions due to the abundant adsorption sites on the surface of Rh nanocrystals, leading to a poor selectivity. As for Rh_1_/CoO, only several stable configurations existed in the single-atom catalyst, so the variety of addition pathways was limited. On the basis of the relative location between H and the nearest unsaturated C in propene, linear products (butyraldehyde) were favoured in configuration I and III, while configuration II and IV were expected to generate branched products (isobutyraldehyde). Due to the small energy difference (0.024 eV) between configuration I and II, the reaction paths deriving from these two configurations were investigated. As revealed in Fig. 4g,h, the whole reaction involves three elementary steps: the addition of one hydrogen atom (step i), the addition of one CO molecule (step ii), and the formation of the product (step iii). On the basis of our calculation, linear and branched products derive from configuration I and II, respectively. Among the elementary steps, step iii is endowed with the highest energy barrier for both configuration I and II, meaning that it is the rate-limiting step (Fig. 4g,h, Supplementary Table 5). The energy barrier of step iii for configuration I is 0.063 eV lower than that for configuration II, indicating that linear products are favoured. In addition, the selectivity of products was also considered in terms of kinetics. Step i and ii were regarded as the quasi-equilibrium states, whereas step iii was the rate-limiting step. At 100 °C, the selectivity for linear products deriving from configuration I and II was calculated as 96%, indicating the dominating product of linear products over Rh_1_/CoO.

## Discussion

In conclusion, we reported a facile synthesis of Rh/CoO which achieved remarkable catalytic performance towards hydroformylation reactions. The configuration of obtained products changed from single atoms to subnanometer clusters and nanoclusters with the increase of Rh mass loading from 0.2% to 1.0% and 4.8%, respectively. In hydroformylation reactions, TOF number of 0.2%Rh/CoO reached 2065, h^−1^, 1.4 and 5.2 times as high as that of 1.0%Rh/CoO and 4.8%Rh/CoO, respectively. In addition, 0.2%Rh/CoO gave the highest selectivity of 94.4% for butyraldehyde. Mechanistic studies revealed that a structural reconstruction of Rh single atoms in Rh/CoO occurred during the catalytic process, facilitating the adsorption and activation of reactants. In terms of a kinetic view, linear products were determined as the dominating products by analysing reaction paths deriving from the two most stable co-adsorbed configurations. This work not only develops an efficient catalyst for hydroformylation reactions with significantly high selectivity, but also advances the understanding of discrepancy in selectivity between single-atom catalysts and traditional metal nanocrystals. As a bridge of homogeneous and heterogeneous catalysis, single-atom catalysts can be potentially applied in other industrial reactions.

## Methods

### Synthesis of CoO nanosheets

CoO nanosheets were synthesized based on the previously reported method^30^. In a typical procedure, 100 mg of Co(acac)_3_ was added into a mixed solution, containing 20 ml of ethylene glycol and 4 ml of distilled water, followed by vigorous stirring for 30 min. Then the mixture was transferred into a 50 ml Teflon-lined autoclave, followed by being sealed and heated at 190 °C for 48 h. After the solution was cooled down to room temperature, the product was collected by centrifugation, washed three times with ethanol and then dried in vacuum overnight.

### Synthesis of Rh/CoO catalysts

In a typical synthesis of 0.2%Rh/CoO, 100 mg of CoO nanosheets and 0.19 ml of Na_3_RhCl_6_ aqueous solution (10 mM) were dissolved in 10 ml of distilled water, followed by stirring at room temperature for 4 h. The product was collected by centrifugation, washed three times with water, and then dried in a vacuum at 50 °C. Further ICP result determined that the mass loading of Rh was 0.2%, well corresponding to the amount of precursors. When the amount of Na_3_RhCl_6_ solution (10 mM) increased to 0.97 and 4.85 ml, 1.0%Rh/CoO and 4.8%Rh/CoO were obtained, where the mass loadings of Rh were 1.0 and 4.8%, respectively. The synthetic procedure of Rh/*bulk*-CoO was similar to that of 0.2%Rh/CoO, except for using commercial CoO to replace CoO nanosheets.

### XAFS measurements

XAFS spectra at Rh K-edge (*E*_0_=23.22 keV) were performed at BL14W1 beam line of Shanghai Synchrotron Radiation Facility (SSRF) operated at 3.5 GeV under ‘top-up' mode with a constant current of 240 mA. The XAFS data on Rh/CoO sample were recorded under fluorescence mode with a Lytle ion chamber or a 32-element Ge solid state detector for different Rh loading samples. The energy was calibrated accordingly to the absorption edge of pure Rh foil. Athena and Artemis codes were used to extract the data and fit the profiles. For the XANES part, the experimental absorption coefficients as function of energies *μ*(*E*) were processed by background subtraction and normalization procedures, which were reported as ‘normalized absorption'. For the EXAFS part, the Fourier transformed (FT) data in *R* space were analysed by applying the 1st shell approximation or metallic Rh model for the Rh–O or Rh–Rh shell, respectively. The passive electron factors, *S*_0_^2^, were determined by fitting the experimental Rh foil data and fixing the Rh–Rh coordination number (*CN*) to be 12, and then fixed for further analysis of the measured samples. The parameters describing the electronic properties (for example, correction to the photoelectron energy origin, *E*_0_) and local structure environment including *CN*, bond distance (*R*) and Debye Waller (*D.W.*) factor around the absorbed atoms were allowed to vary during the fit process.

### Catalytic tests

The hydroformylation of propene was performed in a 100 ml stainless-steel autoclave (Parr Instrument Company). After the addition of 20 ml of isopropanol and catalysts into a Teflon inlet, the autoclave was pressurized with propene (1.6 bar), CO (15.0 bar) and H_2_ (15.0 bar). The amount of catalysts for 0.2%Rh/CoO, 1.0%Rh/CoO and 4.8%Rh/CoO was 50, 10 and 2.1 mg, respectively, to ensure the same amount of Rh species in catalytic tests. The reaction was performed at 100 °C with stirring at 300 r.p.m. for 2 h. After the completion of the reaction, the gas phase was determined by GC-FID. No signals for propane were detected. The liquid phase of the reaction mixture was collected by centrifugation at 10,000 r.p.m. for 2 min, and analysed by GC–MS by using *N*,*N*-dimethylformamide as internal standard. When different olefins other then propene were used as substrate molecules, 5 mmol of olefins, 20 ml of isopropanol and catalysts into a Teflon inlet, the autoclave was pressurized with CO (15.0 bar) and H_2_ (15.0 bar). The reaction was performed at 100 °C with stirring at 300 r.p.m. for 5 h.

### DFT calculations

All the calculations were performed within spin-polarized DFT implemented in VASP^33^. The projector augmented wave (PAW) method^34^ was used to describe the electron–ion interactions. The Perdew–Burke–Ernzerh (PBE) function^35^ was adopted to treat the electron exchange and correlation energy. Hubbard U method with U-J=3.7 was adopted to consider the strong correlated electronic states in CoO as that in previous theoretical work^36^. CoO thin film was simulated by (4 × 4) three Co-O layers with more than 15-Å vacuum layer. Co atoms were considered in ferromagnetic states. The bottom Co-O layer was fixed for the optimization of the configurations and the searching for transition states^35^. The energy cut off was 400 eV and for the large supercell only gamma point is used for *k*-point sampling. The transition states are determined by climbing-image nudged elastic band method^37^, and the searching process did not stop until the force on each atom was less than 0.05 eV/Å.

The reaction paths deriving from configuration I and II were calculated. The whole reaction involves three elementary steps: the addition of one hydrogen atom (step i), the addition of one CO molecule (step ii), and the formation of the product (step iii). In terms of kinetics, step i and ii were regarded as quasi-equilibrium states, whereas the step iii was the rate-limiting step. Thus, the selectivity for linear products deriving from configuration I and II is depicted as *S*_l_*=R*_l_/(*R*_b_*+R*_l_)=(*R*_l_/*R*_b_)/[1+(*R*_l_/*R*_b_)], where *S*_l_, *R*_l_ and *R*_b_ represent the selectivity for linear products, the reaction rate for linear products, and the reaction rate for branched products, respectively. The ratio of the two products is described as *R*_l_*/R*_b_*=*exp(−*E*_a,l−3_/*k*_B_*T*) *θ*_l3−r_/[exp(−*E*_a,b−3_/*k*_B_*T*) *θ*_b3−r_], where *E*_a,l−3_ and *E*_a,b−3_ are the respective activation energies of step iii for linear and branched products, *θ*_l3−r_ and *θ*_b3−r_ are the respective coverages of the reactant in step iii for linear and branched product, *k*_B_ is the Boltzmann constant, and *T* is the reaction temperature. Given the quasi-equilibrium approximation, the ratio of *θ*_l3−r_ to *θ*_b3−r_ can be described as the ratio of the equilibrium constants of the first two elementary steps, and that is *θ*_l3−r_/*θ*_b3−r_*=*exp(−Δ*H*_l−1_/*k*_B_*T*) exp(−Δ*H*_l−2_/*k*_B_*T*)/[exp(−Δ*H*_b−1_*/k*_B_*T*) exp(−Δ*H*_b−2_/*k*_B_*T*)], where Δ*H*_l−1_ and Δ*H*_l−2_ are the respective enthalpies of step i and ii for linear products, while Δ*H*_b−1_ and Δ*H*_b−2_ are the respective enthalpies of step i and ii for branched products. At 100 °C, *S*_l_ is calculated as 96%.

### *In situ* DRIFT tests

*In situ* DRIFT experiments were conducted in an elevated-pressure cell (DiffusIR Accessory PN 041-10XX) with a Fourier transform infrared spectrometer (TENSOR II Sample Compartment RT-DLaTGS) at 100 °C. After flowing 1 atm of N_2_ at the rate of 30 sccm for 0.5 h, the background spectrum of the sample was acquired. Then, 1 atm of H_2_, CO, propene, or the mixed gas (H_2_:CO:propene=1:1:1) were allowed to flow into the cell at 100 °C for 1.0 h. Afterwards, 1 atm of N_2_ was allowed to flow into the cell at 100 °C for 0.5 h. *In situ* DRIFT spectra of the sample after the treatment with different gases were obtained.

### *In situ* XPS measurements

*In situ* XPS measurements were performed at the photoemission end-station at beam line BL10B in the National Synchrotron Radiation Laboratory (NSRL) in Hefei, China. Briefly, the beam line is connected to a bending magnet and covers photon energies from 60 to 1000, eV with a resolving power (*E*/Δ*E*) better than 1,000. The end-station is composed of four chambers, that is, analysis chamber, preparation chamber, quick sample load-lock chamber and high pressure reactor. The analysis chamber, with a base pressure of <2 × 10^−10^ torr, is connected to the beamline and equipped with a VG Scienta R3000 electron energy analyser and a twin anode X-ray source. The high pressure reactor houses a reaction cell where the samples can be treated with different gases up to 20 bar and simultaneously heated up to 650 °C. After the sample treatment, the reactor can be pumped down to high vacuum (<10^−8^ torr) for sample transfer. In the current work, the sample was treated with different gases (1 bar) at 100 °C for 0.5 h in the high pressure reactor, after which it was transferred to analysis chamber for XPS measurement without exposing to air.

### Characterization techniques

HAADF-STEM images were collected on a JEOL ARM-200 F field-emission transmission electron microscope operating at 200 kV accelerating voltage. ICP-AES (Atomscan Advantage, Thermo Jarrell Ash, USA) was used to determine the concentration of metal species. X-ray diffraction patterns were recorded by using a Philips X'Pert Pro Super diffractometer with Cu-Kα radiation (*λ*=1.54178 Å).

### Data availability

The data that support the findings of this study are available from the corresponding authors on reasonable request.

## Additional information

**How to cite this article:** Wang, L. *et al*. Atomic-level insights in optimizing reaction paths for hydroformylation reaction over Rh/CoO single-atom catalyst. *Nat. Commun.* **7,** 14036 doi: 10.1038/ncomms14036 (2016).

**Publisher's note:** Springer Nature remains neutral with regard to jurisdictional claims in published maps and institutional affiliations.

## Supplementary Material

Supplementary Information Supplementary figures and supplementary tables.
