# Supplementary material for: Atomic-level insights in optimizing reaction paths for hydroformylation reaction over Rh/CoO single-atom catalyst
Source: Nat Commun. 2016 Dec 22;7:14036. doi: 10.1038/ncomms14036 (PMC5196038; doi:10.1038/ncomms14036)
Supplement: Supplementary Information — Supplementary figures and supplementary tables. [file ncomms14036-s1.pdf]

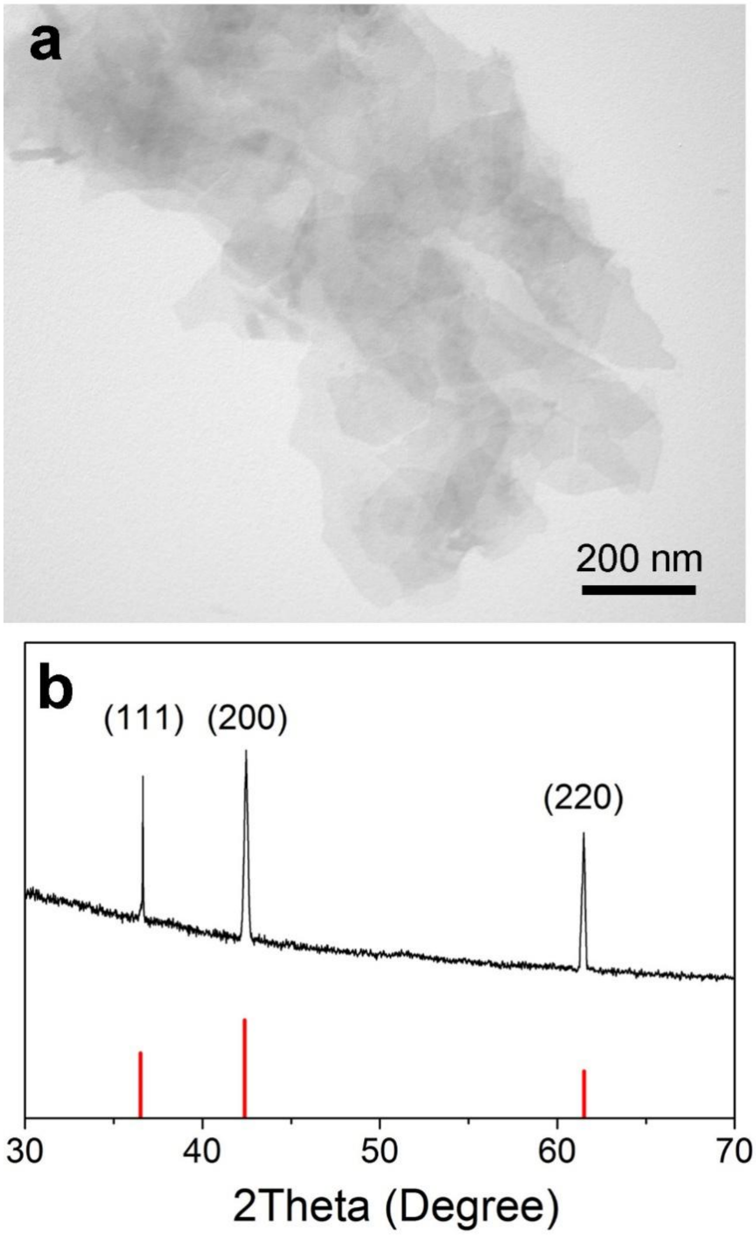

Supplementary Figure 1. (**a**) TEM image and (**b**) XRD pattern of CoO nanosheets.

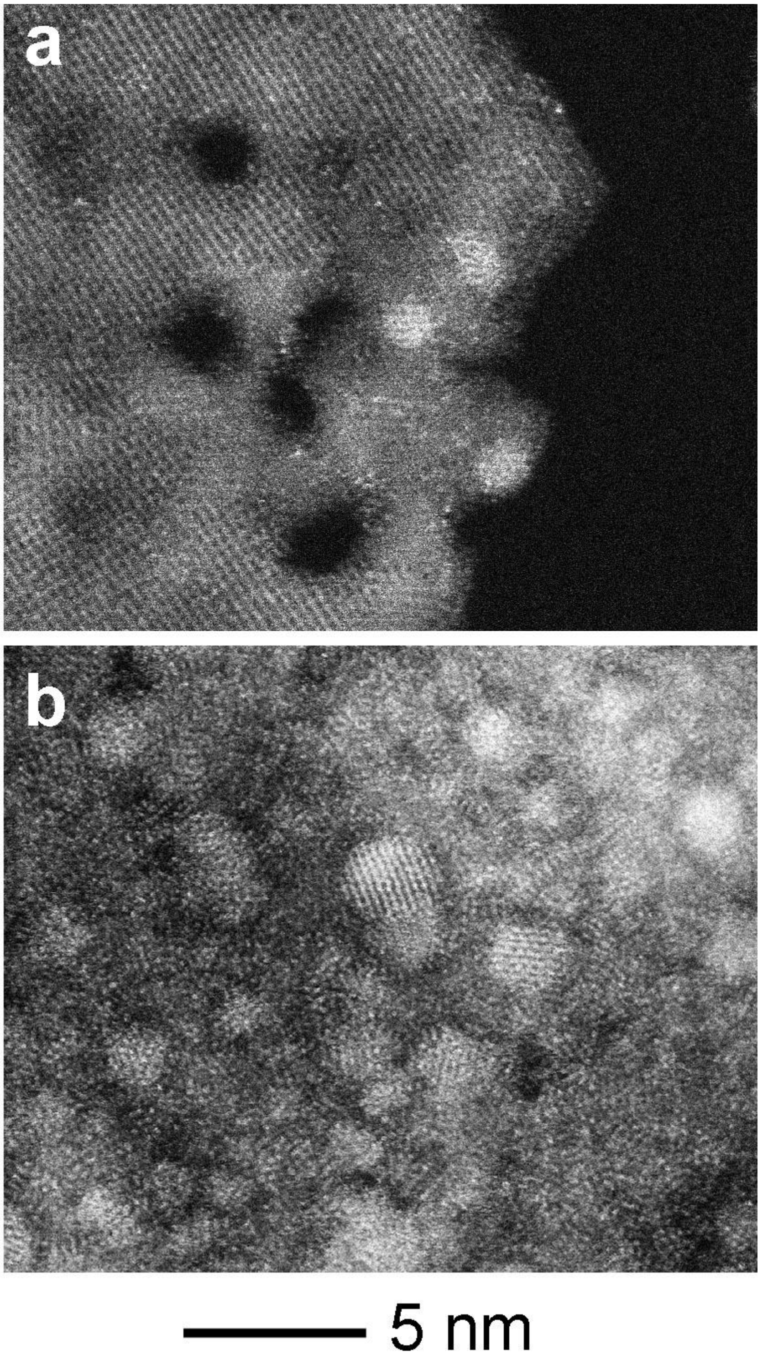

Supplementary Figure 2. HAADF-STEM images of (**a**) 1.0%Rh/CoO and (**b**) 4.8%Rh/CoO.

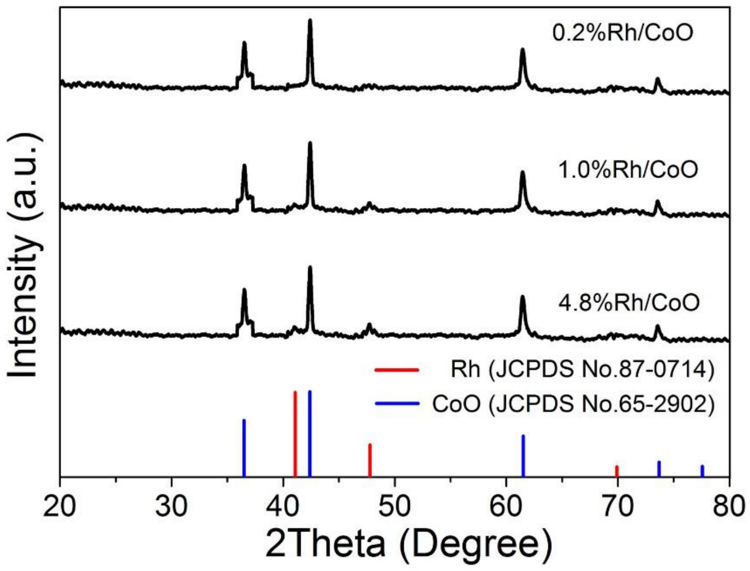

Supplementary Figure 3. XRD patterns of 0.2%Rh/CoO, 1.0%Rh/CoO, and 4.8%Rh/CoO.

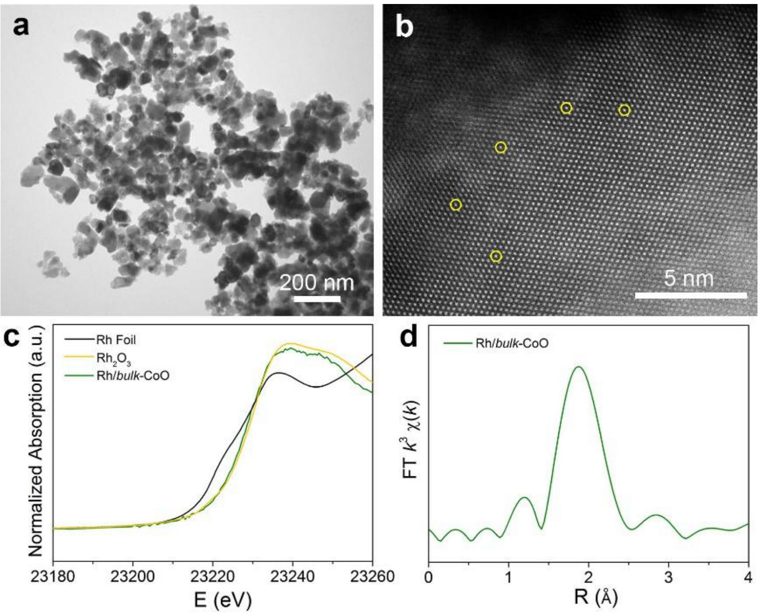

Supplementary Figure 4. Structural characterization of Rh/*bulk*-CoO. (**a**) TEM image of Rh/*bulk*-CoO. (**b**) HAADF-STEM image of Rh/*bulk*-CoO. Rh single atoms marked in yellow circles are uniformly dispersed on the bulk CoO and occupy exactly the positions of Co atoms. (**c**) Rh K-edge XANES profiles for Rh/*bulk*-CoO. (**d**) Rh K-edge EXAFS spectra in *R* space for Rh/*bulk*-CoO.

Supplementary Table 1. EXAFS data fitting results of Rh/CoO with different Rh loadings.

Sample	Rh-O		Rh-Rh		*D. W.*	ΔE_0(eV)
	R (Å)	*CN*	*R* (Å)	*CN*		
Rh foil	—	—	2.69±0.00	12	0.004±0.001	-6.3±1.1
0.2%Rh/CoO	2.04±0.01	5.8±0.6	—	—	0.003±0.001(O)<br>0.005±0.003(Rh)	0.9±0.8
1.0%Rh/CoO	2.02±0.02	4.6±0.8	2.70±0.02	2.6±0.6		
4.8%Rh/CoO	2.02±0.01	4.1±0.6	2.69±0.01	2.5±0.6		

R, distance between absorber and backscatter atoms; *CN*, coordination number; *D. W.*, Debye-Waller factor; ΔE_0, inner potential correction to account for the difference in the inner potential between the sample and the reference compound.

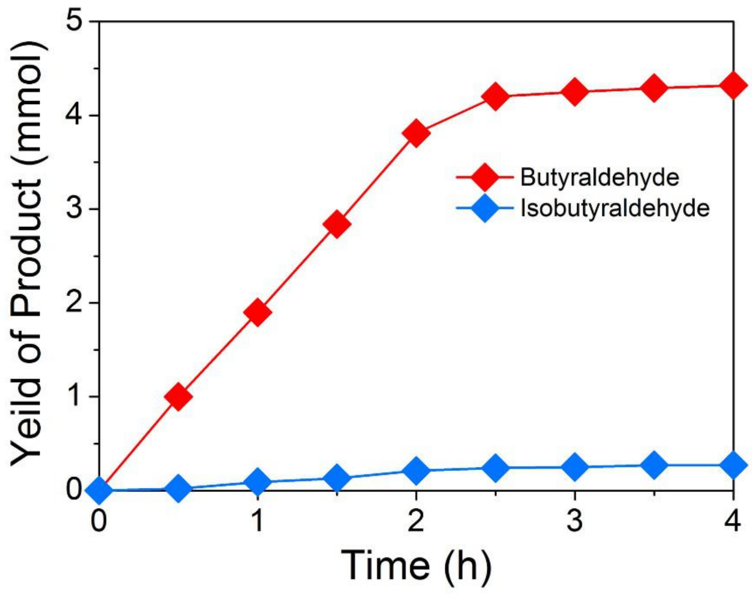

Supplementary Figure 5. Time course of the hydroformylation of propene for Rh/*bulk*-CoO.

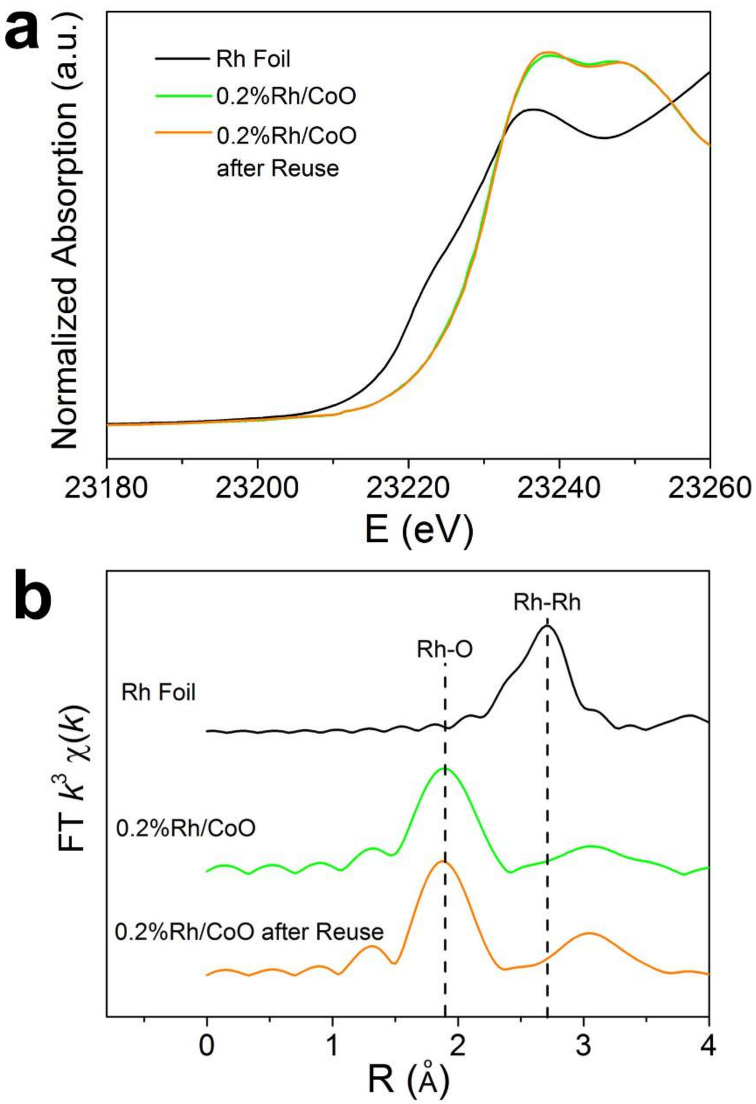

Supplementary Figure 6. (**a**) Rh K-edge XANES profiles for 0.2%Rh/CoO before and after reuse. (**b**) Rh K-edge EXAFS spectra in*R* space for 0.2%Rh/CoO before and after reuse.

Supplementary Table 2. CoO balance and residual Rh species of 0.2%Rh/CoO after different rounds of the reaction.

catalyst	CoO balance (%)	Residual metal (%)
0.2%Rh/CoO after one round	99.8	98.0
0.2%Rh/CoO after five rounds	97.2	96.1

Supplementary Table 3. Catalytic performance of 0.2%Rh/CoO in the hydroformylation reactions by using different olefins at 100 ^{o}C after 5h.

Olefins	Conversion (%)	Selectivity for linear products (%)
Ethylene	91.2	91.6
Heptylene	93.5	90.5
Nonylene	90.7	93.8
Decene	95.8	92.4
Styrene	97.2	86.4

Supplementary Table 4. Adsorption energies of H_2, CO, and propene on Rh_1/CoO.

Adsorbate	Model	Adsorption Energy (eV)
H_2	H_2 on Rh_1/CoO	-0.02
CO	CO on Rh_1/CoO	-1.41
Propene	Propene on Rh_1/CoO	-0.46
2H	OH on Rh_1/CoO	-1.73
Propene	H_2, CO, and propene on Rh_1/CoO	-0.80

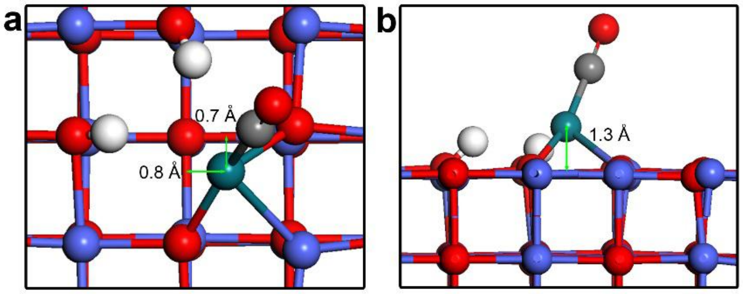

Supplementary Figure 7. (**a**) Top view of the co-adsorbed configuration of both H_2 and CO on Rh_1/CoO, showing the in-plane displacement of the Rh single atom. The Rh single atom deviated from the lattice point by ca. 1.1 Å towards the center of the unit cell. (**b**) Side view of the co-adsorbed configuration of both H_2 and CO on Rh_1/CoO, showing the out-of-plane displacement of the Rh single atom. The Rh single atom moved ca. 1.3 Å out of the plane.

Supplementary Table 5. The reaction enthalpy (*ΔH*, eV) and activation energy (E_a, eV) of each elementary step.

Derived Configuration	Step i		Step ii		Step iii	
Configuration I	ΔH_{l-1}	$E_{a,l-1}$	ΔH_{l-2}	$E_{a,l-2}$	ΔH_{l-3}	$E_{a,l-3}$
	0.378	1.160	0.234	1.032	0.688	1.233
Configuration II	ΔH_{b-1}	$E_{a,b-1}$	ΔH_{b-2}	$E_{a,b-1}$	ΔH_{b-3}	$E_{a,b-3}$
	0.395	1.100	0.25	1.206	0.729	1.296
